# Unraveling the crystal structure of the HpaA adhesin: insights into cell adhesion function and epitope localization of a *Helicobacter pylori* vaccine candidate

**DOI:** 10.1128/mbio.02952-23

**Published:** 2024-02-20

**Authors:** Cyrielle Martini, Victoria Araba, Meriem Beniani, Paula Armoa Ortiz, Mimi Simmons, Mariem Chalbi, Abdelkader Mellouk, Majida El Bakkouri, Charles Calmettes

**Affiliations:** 1 Institut National de la Recherche Scientifique (INRS), Centre Armand Frappier Santé Biotechnologie, Institut Pasteur International Network, Laval, Québec, Canada; 2 National Research Council of Canada (NRC), Human Health Therapeutics Research Center, Montréal, Québec, Canada; 3 PROTEO, the Quebec Network for Research on Protein Function, Structure, and Engineering, Québec city, Québec, Canada; Duke University School of Medicine, Durham, USA

**Keywords:** Helicobacter pylori, adhesins, structural biology, protein structure-function, biophysics

## Abstract

*Helicobacter pylori* is a bacterium that exhibits strict host restriction to humans and non-human primates, and the bacterium is widely acknowledged as a significant etiological factor in the development of chronic gastritis, peptic ulcers, and gastric cancers. The pathogenic potential of this organism lies in its adeptness at colonizing the gastric mucosa, which is facilitated by a diverse repertoire of virulence factors, including adhesins that promote the attachment of the bacteria to the gastric epithelium. Among these adhesins, HpaA stands out due to its conserved nature and pivotal role in establishing *H. pylori* colonization. Moreover, this lipoprotein holds promise as an antigen for the development of effective *H. pylori* vaccines, thus attracting considerable attention for in-depth investigations into its molecular function and identification of binding determinants. Here, we present the elucidation of the crystallographic structure of HpaA at 2.9 Å resolution. The folding adopts an elongated protein shape, which is distinctive to the *Helicobacteraceae* family, and features an apical domain extension that plays a critical role in the cell-adhesion activity on gastric epithelial cells. Our study also demonstrates the ability of HpaA to induce TNF-α expression in macrophages, highlighting a novel role as an immunoregulatory effector promoting the pro-inflammatory response *in vitro*. These findings not only contribute to a deeper comprehension of the multifaceted role of HpaA in *H. pylori* pathogenesis but also establish a fundamental basis for the design and development of structure-based derivatives, aimed at enhancing the efficacy of *H. pylori* vaccines.

**IMPORTANCE:**

*Helicobacter pylori* is a bacterium that can cause chronic gastritis, peptic ulcers, and gastric cancers. The bacterium adheres to the lining of the stomach using proteins called adhesins. One of these proteins, HpaA, is particularly important for *H. pylori* colonization and is considered a promising vaccine candidate against *H. pylori* infections. In this work, we determined the atomic structure of HpaA, identifying a characteristic protein fold to the *Helicobacter* family and delineating specific amino acids that are crucial to support the attachment to the gastric cells. Additionally, we discovered that HpaA can trigger the production of TNF-α, a proinflammatory molecule, in macrophages. These findings provide valuable insights into how *H. pylori* causes disease and suggest that HpaA has a dual role in both attachment and immune activation. This knowledge could contribute to the development of improved vaccine strategies for preventing *H. pylori* infections.

## INTRODUCTION

*Helicobacter pylori* is a gram-negative, microaerophile bacteria that selectively colonize the surface of the gastric mucosa in both human and non-human primates. Currently, the global prevalence of *H. pylori* infection exceeds 50%, with considerable variation ranging from 10% to 70% depending on factors such as ethnicity, age, and socioeconomic status (1–4). If left untreated, *H. pylori* colonization persists lifelong and can lead to a range of gastric pathologies, including gastritis, peptic ulcers, and various types of gastric cancers (5–7). Accordingly, the bacterium is categorized as a class 1 (definite) carcinogen by the World Health Organization emphasizing its prominent role in the development of gastric cancers. The precise molecular mechanisms underlying gastric carcinogenesis following *H. pylori* infections are not fully understood but involve multiple virulence factors that disrupt key signaling pathways within the host, thereby promoting a neoplastic state of growth (8). The burden of *H. pylori*-related diseases is very substantial, with gastric cancer ranking as the sixth most diagnosed cancer and the fourth leading cause of cancer-related deaths worldwide, underscoring the importance of eradicating *H. pylori* infections (9).

The adhesion of *H. pylori* is regarded as a crucial initial step in the pathogenesis of the bacterium within the stomach (10, 11). Despite the challenges posed by gastric peristalsis and the presence of a protective mucus covering the gastric mucosa, *H. pylori* has evolved into a successful human colonizer by establishing robust interactions with gastric epithelial cells. The bacterium uses flagellar motility to navigate through the mucus layer, which consists of heavily glycosylated mucin proteins, ultimately attaching to the epithelial cells situated beneath it (8, 12). *Helicobacter pylori* possess a wide range of adhesion factors predominantly found in the outermembrane, which facilitate interactions with cell host receptors (CEACAM, integrin), components of the extracellular matrix (laminin), or glycans (Lewis antigens) (13, 14). The expression and regulation of these adhesins in *H. pylori* exhibit intricate patterns, including phase variation and extensive allelic diversity between clinical isolates, suggesting that the bacterium modulates its surface properties to rapidly adapt to individual hosts (15, 16).

*H. pylori* adhesin A (HpaA) stands out as a pivotal adhesin that plays an indispensable role in the successful establishment of colonization by the pathogen (17). Originally characterized as a neuraminyllactose-binding hemagglutinin (18), HpaA is a 26 kDa lipoprotein found on the bacterial surface and flagellar sheath (19, 20). The gene encoding HpaA is prevalent in most *H. pylori* strains, and its amino-acid sequence displays a high degree of conservation among *H. pylori* isolates. Remarkably, these proteins appear to be exclusive to the Helicobacteriaceae family, as they do not exhibit significant sequence homologies with proteins from other taxa (21, 22). Similar to other notable *H. pylori* proteins, such as Urease (UreA or UreB), Cytotoxin-associated gene A (CagA), Vacuolating cytotoxin A (VacA), HpaA is also highly immunogenic (23). For these reasons, HpaA is considered a promising vaccine candidate against *H. pylori*. Antibodies against HpaA are detected in the sera of both symptomatic and asymptomatic patients infected with *H. pylori* (24). Therefore, the protein has been incorporated into various vaccine formulations, either as a full-length protein or as peptide fragments, which have demonstrated the ability of HpaA antigens to elicit strong immune responses in the stomach and reduce bacterial colonization in animal models (25–28).

Despite extensive research efforts, the precise mechanism by which HpaA coordinates bacterial adhesion remains elusive. In its early discovery, HpaA was initially categorized as a neuraminyllactose-binding hemagglutinin due to the presence of a putative motif (residues _107-_KRTIQK_-112_) that bears approximate resemblance to a sialic acid-binding motif (KARAVASK) identified in the fimbriae subunit SfaS of *Escherichia coli* (18, 29). However, conflicting experimental data has raised doubts regarding the sialic acid-binding function of HpaA, as recombinant HpaA proteins failed to discriminate between sialylated and non-sialylated proteins in ELISA assays (30). Based on local sequence comparison with the Shiga-like toxin 1, a bioinformatic study has proposed an alternate hypothesis, suggesting that HpaA may engage interactions with glycolipids through a putative lactosylceramide-binding motif (residues _112-_KKSEPGLLFSTGLDK_-126_) (31). More recently, another study has put forth the idea that annexin A2 could potentially serve as a receptor for HpaA, a premise supported by the co-elution of Annexin A2 and other host proteins with HpaA in a proteomic setup (32). However, these hypotheses lack experimental validation, necessitating further comprehensive investigations to uncover the native receptor of HpaA and elucidating the binding determinants that governs its molecular interactions with the host.

To deepen our understanding of the molecular mechanisms underlying the adhesin activity of HpaA, we conducted a structure-activity relationship study, elucidating key-binding contributors of the adhesin-host recognition. Notably, we resolved the first crystallographic structure of HpaA at a resolution of 2.9 Å, revealing a variable domain within the neuraminyllactose-binding hemagglutinin family. This domain plays a critical role in cell-adhesion to gastric epithelial cells in a sialic-acid-independent manner, with the identification of two important binding motifs cross-validated by site-directed mutagenesis and functional assays. Moreover, this study unveils a novel immunoregulatory function of HpaA, as the adhesin induces the expression of TNF-α cytokines in macrophages, suggesting a multifaceted role of HpaA during *H. pylori* infection. The structural insights gained from the HpaA protein additionally offer valuable information regarding the localization of epitopes. Altogether, these findings not only enhance our comprehension of HpaA’s molecular function but also open new avenues for the rational design of improved immunogens capable of eliciting enhanced immune responses, guiding future vaccine development efforts.

## RESULTS

### Sequence and structural comparisons of HpaA reveal a protein family restricted to Helicobacteraceae

**Fig 1:**
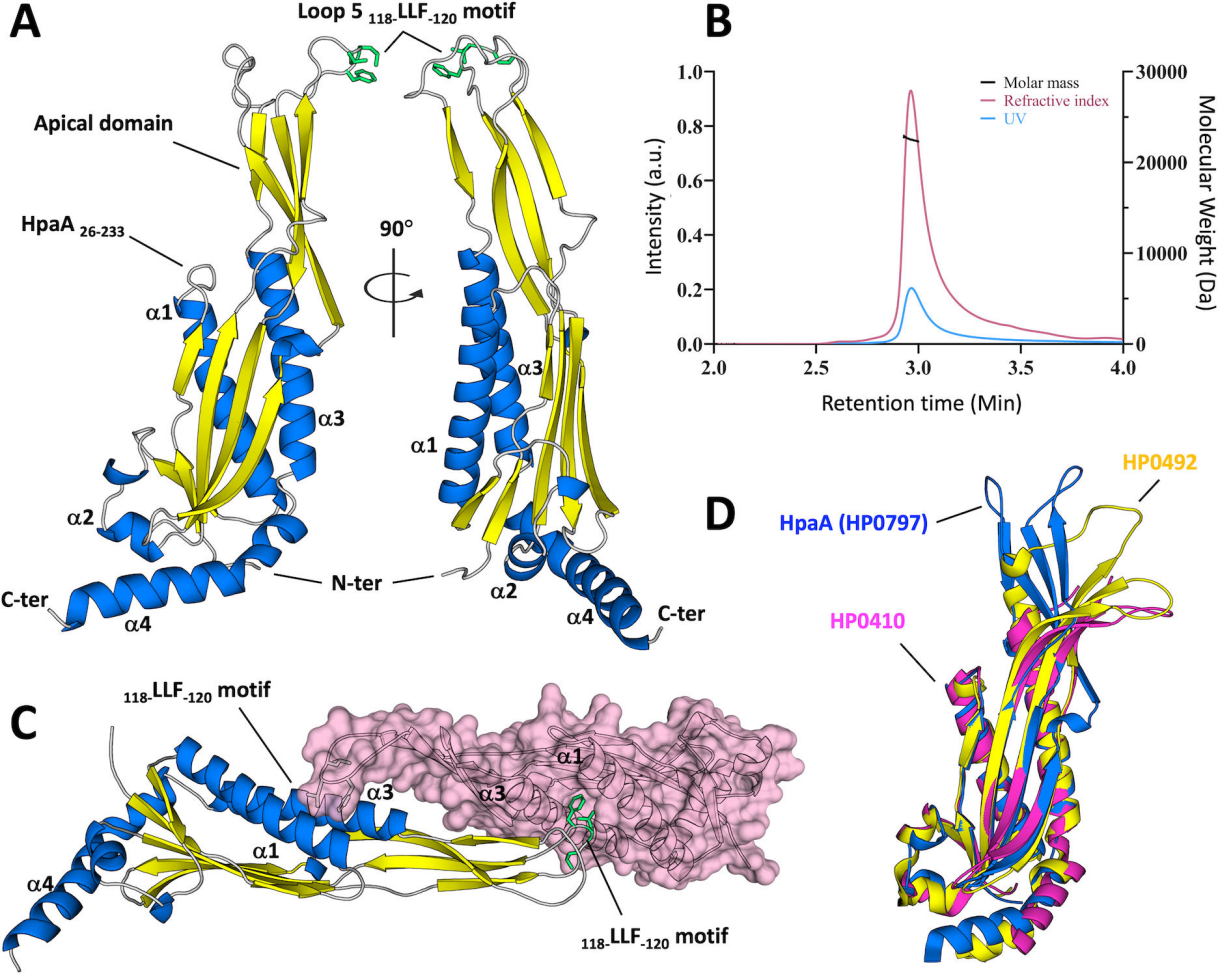
Structural snapshots of HpaA. (**A**) Side views of the HpaA_26–233_ crystal structure are depicted in a cartoon representation. The structure is color-coded to highlight the secondary structural elements, with β-sheets shown in blue and α-helices in yellow. The side chains of the _118-_LLF_-120_ motif (loop 5) are visualized in green using a stick representation. (**B**) The oligomerization state of HpaA_26–233_ was determined by multi-angle laser light scattering (MALLS). The single elution peak corresponds to the monomeric form of HpaA in solution. The average molecular weight is calculated to be 22.5 kDa. (**C**) In the crystal lattice, the four HpaA molecules from the asymmetric unit adopt a swapped-domain dimeric arrangement. Each _118-_LLF_-120_ loop 5 motif is buried between the helices α1 and α3 of the adjacent protein. (**D**) Structural alignment with HP0410 and HP0492, two structural homologs identified from the pdb entries 3BGH and 2I9I by PDBeFold (33). HpaA, HP0410, and HP0492 are colored in blue, magenta, and yellow, respectively.

The crystal structure of HpaA_26–233_ was determined using a C-terminally his-tagged protein construct, excluding the first 25 amino acids from the mature lipoprotein (Fig. 1). Initial attempt to crystallize the full-length HpaA_2-233_ was unsuccessful, leading to the engineering of the HpaA_26–233_ construct resolved at 2.9 Å resolution. The truncated amino terminal sequence of 25 residues is predicted to be a flexible peptide that anchors the lipoprotein to the bacterial surface via a conserved amino-terminal lipid-modified cysteine residue. This allows the globular domain to project above the bulky layer of lipopolysaccharide (LPS) surrounding the bacterial cell. The protein crystallized as two homodimers within the asymmetric unit of the P2_1_ space group (Fig. 1C; Fig. S1), a conformation that likely results from the large crystal lattice and tight packing of the protein within the crystal, with 40% solvent (Table 1). Further characterization of the molecule using multi-angle light scattering confirmed that HpaA_26-233_ exists as a homogeneous and monomeric species in solution, with a calculated molecular mass of 22.5 kDa (Fig. 1B).

**TABLE 1:** Refinement statistics for the HpaA_26–233_ crystal structure

	HpaA _26–233_
PDB code	8T8D
Phasing method	Molecular replacement
Data collection^*a*^	
Space group	P 1 2_1_ 1
Cell dimensions:	
*a*, *b*, *c* (Å)	82.5, 59.8, 94.0
a, b, c (°)	90, 100.85, 90
Wavelength (Å)	0.95375
Resolution (Å)	48.1–2.9 (3.0–2.9)
Total reflections	138,948 (14,418)
Unique reflections	20,236 (2,002)
*I* / σ*I*	12.5 (2.8)
Completeness (%)	99.6 (99.6)
Redundancy	6.9 (7.2)
R-merge	0.23 (0.82)
CC_1/2_	0.99 (0.91)
Refinement^*a*^	
Resolution (Å)	48.1–2.90
*R*_work_ / *R*_free_	0.24/0.30
No. of atoms	6,369
Protein	6,280
Glycerol/ions	42
Water	67
*B*-factors	
Protein	82.9
Ligands	145.6
Water	58.1
R.m.s. deviations	
Bond lengths (Å)	0.006
Bond angles (°)	0.97
Ramachandran	
Favored (%)	96.2
Outlier (%)	0.3
Clashscore	8.8

^*a*^ Highest resolution shell is shown in parenthesis.

**Fig 2:**
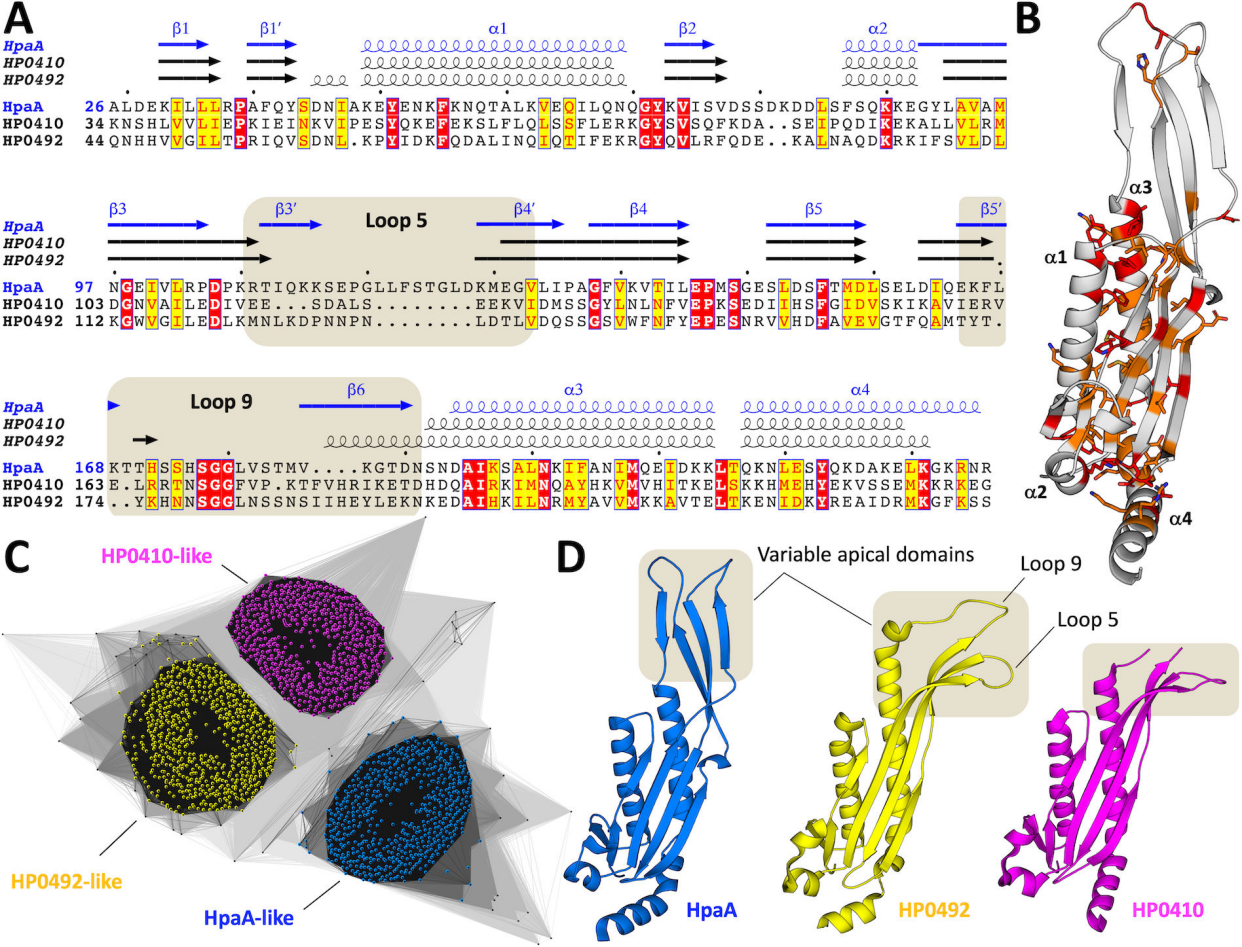
Structural alignment and cluster sequence analysis of the Neuraminyllactose-binding hemagglutinin superfamily. (**A**) Sequence alignment of HpaA, HP0410, and HP0492. The secondary structure elements and loops from the apical domain are shown above the sequences. Identical residues and conserved residues are highlighted and boxed in red and yellow, respectively. The apical domain is indicated with a brown background. (**B**) The sequence conservation information is mapped onto the HpaA structure. Identical residues and conserved amino acids are colored in red and orange, providing insight into region of significant conservation within the protein. (**C**) To assess the broader context of HpaA, all sequences from the Neuraminyllactose-binding hemagglutinin superfamily (PFAM entry PF05211) were extracted and subjected to sequence-based classification using CLANS (34). The protein family comprises 2002 sequences, 1990 of them originating from the *Helicobacter* genus. The resulting plot depicts an all-against-all pairwise BLAST clustering of individual sequences in a two-dimensional space. Line connections are drawn between similar sequences based on a *P*-value cut-off of 1e^−2^, and the line distances represent proportional sequence similarities. The majority of sequences cluster into three distinct groups represented by HpaA, HP0410, and HP0492. These groups are not mutually exclusive and can coexist within *helicobacter* genomes. (**D**) Structural representations of HpaA, HP0410, and HP0492 are shown in the same orientation. The apical domain, which corresponds to the most variable region of the protein family in term of both sequence and structure, is highlighted and boxed in brown.

The crystal structure reveals that HpaA is an elongated protein consisting of two successive antiparallel β-sheets, with an apical extension protruding from a more globular domain composed of one of the β-sheets surrounded by three α-helices (α1-α3-α4) stabilized together by hydrophobic interactions. This folding pattern is atypical and has only been observed in two other proteins in the Protein Data Bank (Fig. 1D). Accordingly, structural homology searches have only identified two uncharacterized lipoprotein homologs from *H. pylori*, namely, HP0410 and HP0492, which share sequence identities of 23% and 24% and root-mean-square deviations (r.m.s.d.) of 1.2 Å and 1.4 Å with HpaA, respectively (Fig. 2A and B). Further sequence analysis using the PFAM protein families database reveals that the Neuraminyllactose-binding hemagglutinin superfamily, which includes HpaA, HP0410, and HP0492, is exclusively found in *Helicobacteraceae* genomes (Fig. 2C). The protein family clusters into three distinct groups, represented by HpaA, HP0410, and HP0492 that coexist within the same *H. pylori* genomes.

The overall fold is well conserved among the three homologs, except for the apical domain, which exhibits significant structural and sequence variations (Fig. 2). This alludes to a specialization of the apical domain for specific functions or binding partners. Of particular interest, the apical loop L_5_ harbors an intriguing feature in the form of a hydrophobic _118-_LLF_-120_ motif that is fully exposed to the aqueous environment. It is anticipated that such surface-exposed hydrophobic motif would reduce its solvent accessibility and contribute to interactions with other constituents. Consistently, all four _118-_LLF_-120_ motifs observed in the asymmetric unit are engaged in similar crystal contacts with adjacent HpaA molecules, burying the leucine and phenylalanine residues within a neighboring cavity formed between the α-helices α1 and α3 (Fig. 1C; Fig. S1).

### HpaA binds to AGS cells in a neuraminic acid-independent manner

**Fig 3:**
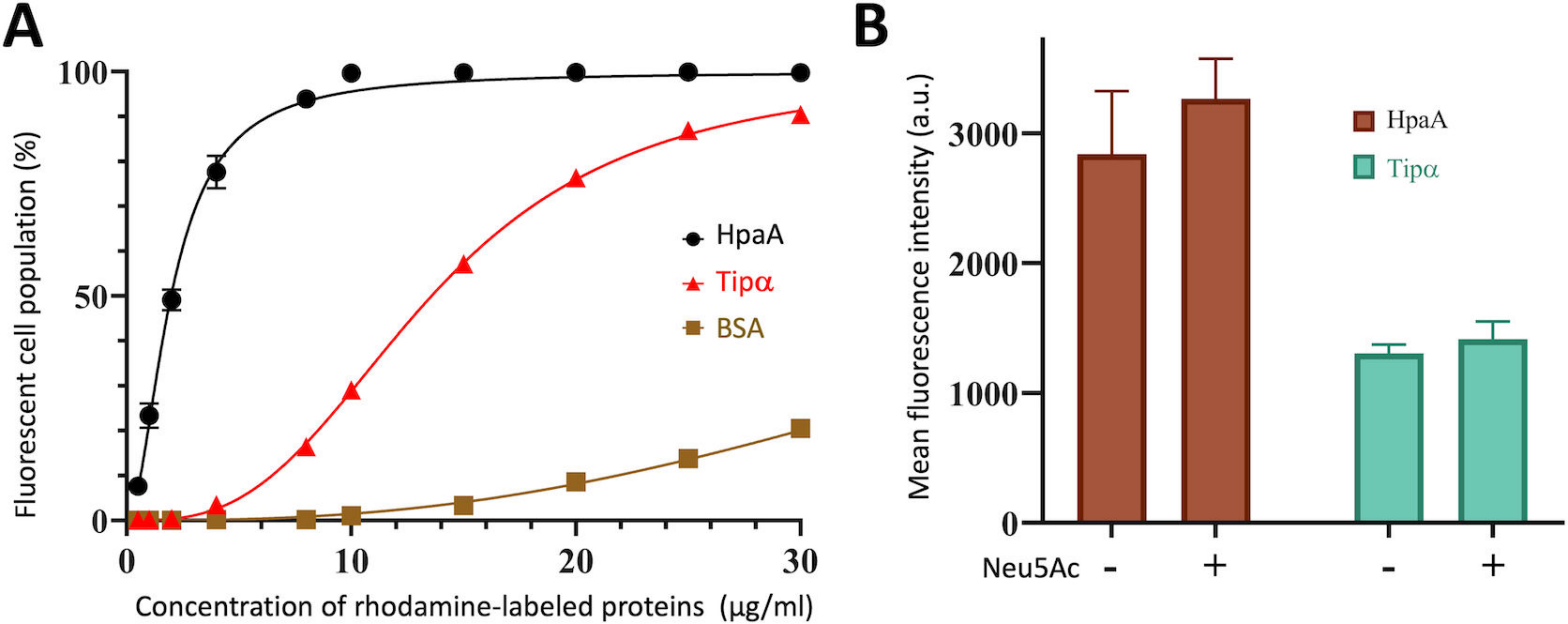
HpaA binds to AGS cells in a neuraminic acid-independent manner. (**A**) Dose-response curves depict the binding titration of rhodamine-coupled HpaA_26–233_, Tipα (positive control), and bovine serum albumin (negative control) with AGS cells. The binding interaction was monitored by flow cytometry using biological triplicates. (**B**) Histograms depict the binding association of HpaA_26–233_ and Tipα (positive control) rhodamine-coupled proteins with AGS cells in the absence and presence of saturating concentration of neuraminic acid at 800 µg/mL. AGS cells were incubated with 4 and 20 µg/mL of HpaA_26–233_ and Tipα, respectively, followed by three washes prior to quantification of cellular fluorescence using flow cytometry. The histogram represents the means and standard deviations derived from three independent experiments. The ANOVA test reveals no statistical difference in the binding competitive assays of HpaA and Tipα (*P*-values of 0.22 and 0.88, respectively).

**Fig 4:**
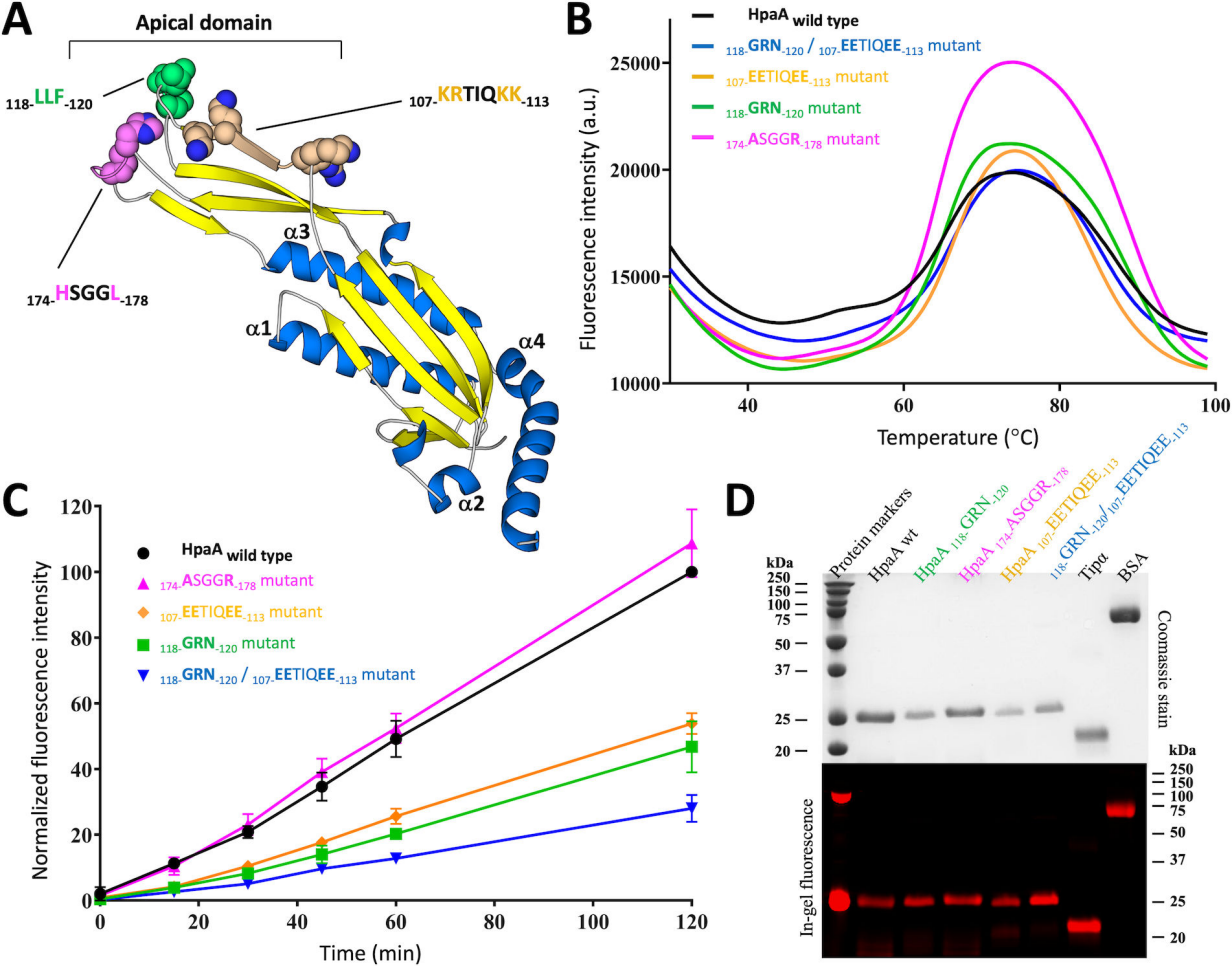
HpaA binding site is located on the apical domain. (**A**) Mapping of the amino acids subjected to site-directed mutagenesis onto the HpaA_26–233_ crystal structure. The mutated side chains are depicted using a sphere representation. The labeled sequences correspond to the wild type, while the locations of the point mutations are color-coded in green, magenta, and orange. The color code is kept consistent in other panels. (**B**) Measurement of thermal denaturation of wild type and mutant HpaA_26–233_ proteins was conducted by differential scanning fluorescence. The similar melting temperature values (73°C) between the wild-type and mutant proteins confirm that the selected point mutations do not alter protein folding and stability. (**C**) Time course of the binding association between AGS cells and rhodamine-labeled HpaA proteins (4 µg/mL) monitored by flow cytometry. The binding association is reported for wild-type and mutated HpaA_26–233_ constructs. The plot traces the mean values and standard deviations calculated from three biological replicates. (**D**) SDS PAGE analysis shows the migration of recombinant HpaA_26–233_ proteins, Tipα, and BSA following amine coupling reaction to rhodamine fluorescent dyes. The proteins are visualized using Coomassie staining and in-gel fluorescence.

HpaA was originally classified as a Neuraminyllactose-binding hemagglutinin in 1993 (18). However, the assigned function has been a subject of controversy due to conflicting observations obtained from intricate and heterogeneous samples (live bacteria or cell lysates), which are susceptible to pleiotropic effects. To overcome these challenges, we capitalized on the production of a high-quality recombinant protein and developed a direct binding assay between HpaA and AGS cells, a human gastric epithelial cell-line (Fig. 3). For this assay, the proteins were covalently linked to a rhodamine (RhB) fluorescent dye via an amine coupling reaction, with a final molecular labeling ratio of 1:1.5 (protein/dye). The labeling ratio was optimized to ensure uniform distribution of the fluorophore across the labeled proteins and to prevent protein inactivation caused by excessive labeling (the protein surface displays 31 lysines and arginines that are evenly distributed). Subsequently, the labeled protein was purified by size exclusion chromatography to eliminate free dyes and collect homogeneous RhB-HpaA samples (Fig. 4D). The adhesin activity was assessed by flow cytometry to monitor the direct association of RhB-HpaA with AGS cells. The binding activity was compared to that of the Tipα protein, a secreted *H. pylori* effector known to associate with cell surface nucleolin receptors, serving as a positive control. Flow cytometric analysis demonstrated a robust association of HpaA with AGS cells, displaying higher binding association compared to the Tipα effector (Fig. 3).

To further investigate the neuraminyllactose-binding hemagglutinin function, we conducted a competition assay using saturating concentrations of exogenous sialic acid. However, despite the presence of sialic acid in high concentration, we were unable to reduce HpaA-binding activity with AGS cells, suggesting that HpaA likely interacts with a yet unidentified receptor on the host cells. In an effort to identify the specific target receptor, we also examined binding of HpaA with mucin, a heavily glycosylated protein and major component of the gastric mucosa, as well as Annexin A2, which was identified in a recent proteomic analysis. However, using competition binding assays, we were unable to detect any binding activity between HpaA and glycosylated mucin or Annexin A2 (Fig. S2).

### HpaA associates to AGS cells using an apical hydrophobic loop

The structural characterization of HpaA presents an exceptional opportunity to investigate the contribution of specific structural motifs to its adhesion activity (Fig. 4; Fig. S3). For the purpose of studying this structure-activity relationship, we targeted three conserved protein motifs: the apical motifs _118-_LLF_-120_ (loop L_5_) and _174-_HSGGL_-178_ (loop L_9_) which were mutated to _118-_GRN_-120_ and _174-_ASGGR_-178_, respectively, as well as the _107-_KRTIQKK_-113_ motif, which was mutated to _107-_EETIQEE_-113_ (Fig. S4). The selection of the apical loops L_5_ and L_9_ was based on their localization within the most divergent structural elements of the protein family, and the _107-_KRTIQKK_-113_ motif was chosen due to its suggested involvement in host cell interactions (18, 35). Our mutation strategy aimed to modify the biophysical properties of these motifs by introducing charge reversal mutations or altering the hydrophobicity index. To ensure the reliability of our results and avoid potential distortions caused by protein instability, we performed a comprehensive validation of the protein constructs by differential scanning fluorescence (Fig. 4B). This validation confirmed that the mutant proteins exhibit similar solution properties and thermostability profiles, indicating comparable behavior to the wild-type protein. The adhesin function of the mutants was ultimately quantified using flow cytometry and compared to wild-type HpaA to evaluate their binding association with AGS cells (Fig. 4C).

According to the cell adhesion assays, the flow cytometric analysis unveils the significant contribution of the _118-_LLF_-120_ apical loop L_5_ to host-cell recognition, as mutation within this loop resulted in a 55% decrease in binding association with AGS cells. In contrast, alteration within the second apical loop L_9_ (_174-_HSGGL_-178_ motif) did not affect cellular adhesion. Furthermore, we reaffirmed the importance of the _107-_KRTIQKK_-113_ motif as a binding determinant and identified the critical role of lysine and arginine side chains in maintaining efficient adhesion. Interactions mediated by this motif were severely impeded by reverse charge mutations (_107-_EETIQEE_-113_ mutants) leading to a substantial loss of binding activity with a 50% decrease observed (Fig. 4C; Fig. S3). Notably, the accumulation of mutation in both the _118-_LLF_-120_ and _107-_KRTIQKK_-113_ regions did not exhibit cumulative effects but resulted in a more pronounced phenotype. This suggests that these sites interact with the same receptor, forming a large binding interface on the apical domain of HpaA.

### HpaA promotes TNF-α expression in macrophages

**Fig 5:**
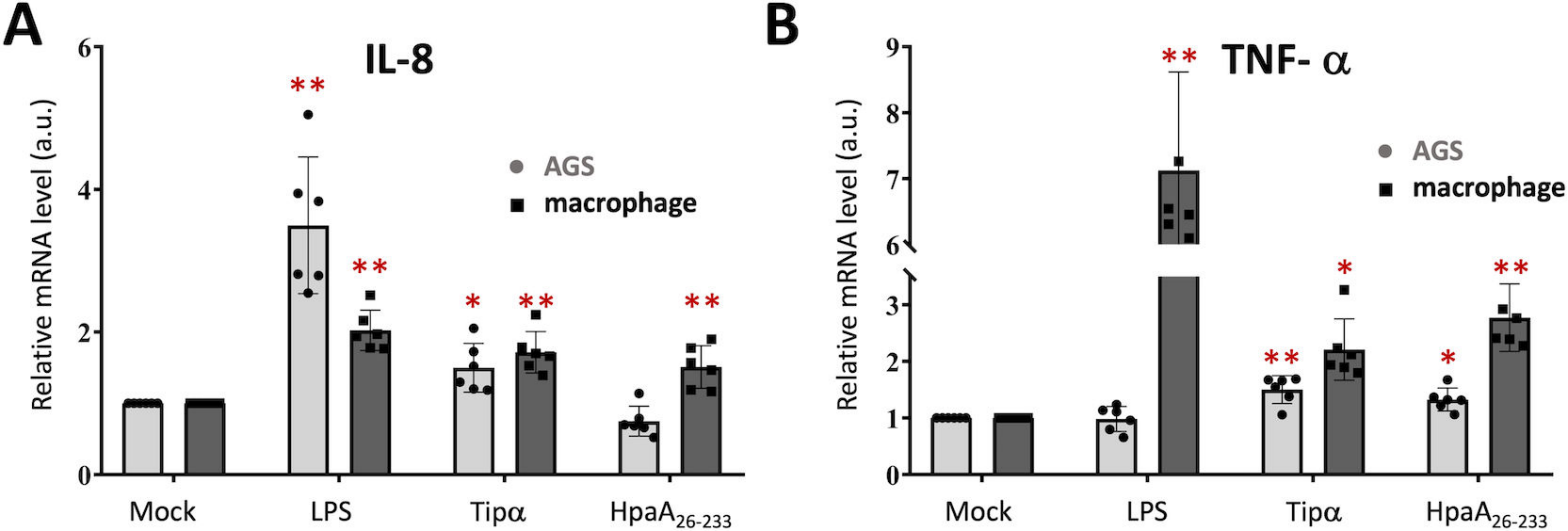
HpaA promotes TNF-α proinflammatory response in macrophage. (**A**) Quantification of IL-8 mRNA levels through real-time RT-PCR analysis in AGS and macrophage cells, comparing treated and untreated samples. (**B**) Quantification of TNF-α mRNA levels through real-time RT-PCR analysis in AGS and macrophage cells, comparing treated and untreated samples. AGS cells and THP-1-derived macrophages were incubated with the eluted protein buffer, 10 µg/mL LPS (lipopolysaccharide), 100 µg/mL Tipα (TNF-α inducing protein), and 50 µg/mL HpaA_26-233_ for 4 h prior mRNA extraction. LPS and Tipα were used as positive controls. The threshold cycles (*Ct* values) were normalized to their corresponding GADPH mRNA, and the comparative mRNA levels were determined using the 2^−ΔΔ*Ct*^ method. The histograms depict the mean values and standard deviations calculated from two biological duplicates. Red asterisks denote statistical significances of HpaA, LPS, and Tipα excitations compared to untreated samples as determined by a one-way analysis of variance (ANOVA) test (*P ≤ 0.05, **P ≤ 0.005).

The adhesion of *H. pylori* to host cells has been established to influence host cell signaling and cytokine production, a process that involves various bacterial effectors such as the CagL adhesins or Tipα effectors (36–38). However, the cytokine response specifically triggered by HpaA remains elusive. To address this gap, we leveraged our experimental set up to examine the gene expression pattern of proinflammatory cytokines in macrophages and AGS cells following their *in vitro* interaction with HpaA, subsequent to validating HpaA’s binding to macrophages (Fig. S5). We monitored the mRNA expression of commonly reported cytokines, such as IL8 and TNF-α, which are associated with *H. pylori* infection (Fig. 5). The culture supernatants of THP-1-derived macrophages and AGS cells were incubated with HpaA for 4 h and compared to control cells using quantitative RT-PCR (unstimulated negative control; LPS and Tipα positive control reactions). Our data establishe that HpaA stimulation results in a 1.3-fold increase in TNF-α cytokine induction in AGS cells and a 2.8-fold increase in macrophage cells. Significantly, this response closely mirrors the positive control reaction triggered by the TNF-α inducing protein, Tipα, a bacterial effector utilized by *H. pylori* to sustain chronic inflammation in gastric tissues (39). Furthermore, we detect additional IL8 responses in macrophages upon HpaA stimulation, demonstrating once again comparable response to the Tipα effector. These findings suggest that HpaA may play a role not only as a surface adhesin facilitating *H. pylori* adherence to host cells but also as a modulator of the host cell signaling, contributing to the establishment of a favorable microenvironment.

### Identification of immunogenic epitopes on the HpaA structure

**Fig 6:**
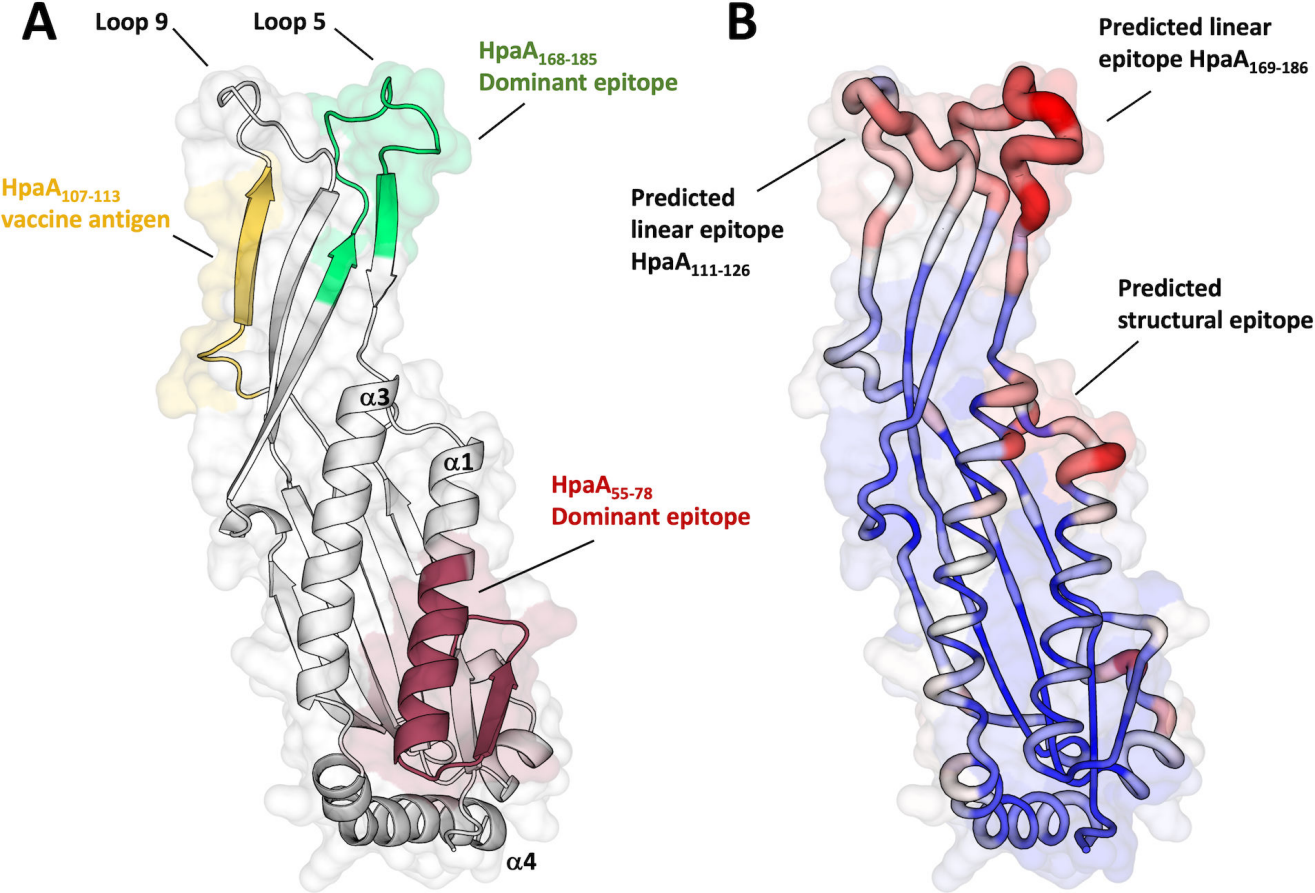
Mapping epitopes on HpaA. (**A**) Mapping of current HpaA peptide antigens (25, 26, 44–46) and previously identified immunodominant epitopes (43) onto the HpaA structure. (**B**) Structure-based prediction of B cell epitopes using ScanNet (42), a geometric deep learning model. The predicted epitopes are mapped onto the HpaA crystal structure; the confidence scores are represented with a color gradient ranging from blue (low score) to red (high score).

In addition to its essential role for the colonization of *H. pylori*, HpaA is a conserved, immunogenic protein located on the bacterial surface, considered a promising antigen for therapeutic vaccines against *H. pylori* (40). As such, previous studies have demonstrated the efficacy of recombinant HpaA antigens and derived peptide epitopes in reducing bacterial colonization in murine infection models (41). Building upon the structural elucidation of HpaA, we aimed to map known immunodominant regions and currently used vaccine epitopes onto the surface of the HpaA structure, providing a rationale for the future design of improved immunogens (Fig. 6). Moreover, this structural information opens new avenues for identifying unexplored antigenic epitopes. To this end, we employed a structure-based prediction method using ScanNet (42), a deep learning model, to computationally predict putative B-cell epitopes that could further enhance HpaA vaccination strategies. The predicted B-cell epitopes, illustrated in Fig. 6B, delineate three regions of interest consisting of two linear epitopes and one structural epitope. Notably, the suggested HpaA_124–139_ linear epitope overlaps with the functionally important _118-_LLF_-120_ apical loop, making it a strong candidate, as antibodies targeting this region are expected to inhibit the critical adhesin activity of HpaA (17). The other relevant epitopes encompass the second apical loop, spanning the HpaA_183–199_ regions, which coincides with a previously reported immunodominant site identified in CD4^+^ T-cells derived from *H. pylori*-infected patients (43), as well as a structural motif located at the junction of the α-helices α1 and α3.

## DISCUSSION

The results presented in this study provide valuable insights into the structural characteristics, binding properties, and immunogenicity of the HpaA adhesin from *H. pylori*. The crystal structure of HpaA_26–233_, resolved at 2.9 Å resolution, reveals an elongated-shape-protein consisting of two antiparallel β-sheets that define an apical extension critical for the cell-adhesion activity of HpaA. Previous studies have classified HpaA as a neuraminyllactose-binding hemagglutinin (18, 29); however, conflicting observations have led to controversies regarding this function. To address this ambiguity and gain clarity on the role of HpaA, we capitalized on the purification and labeling of homogeneous HpaA protein samples to conduct direct binding assays between HpaA and AGS cells. Our data illustrate the remarkable binding capacity of HpaA to AGS cells, surpassing even the cell association levels of Tipα, a well-known secreted effector of *H. pylori* that interacts with surface nucleolin receptors (47). In relation to the debated neuraminyllactose binding activity, our findings indicate that the association of HpaA with AGS cells is not reliant on neuraminic acid. This conclusion is supported by the fact that competition with exogenous sialic acid does not reduce the binding activity, suggesting the involvement of a receptor other than neuraminic acid in mediating host-cell interactions with HpaA adhesins.

To investigate the underlying factors responsible for the binding activity of HpaA, site-directed mutagenesis was employed to target specific motifs within the apical domain of HpaA. Combined with a quantitative flow cytometry-binding assay, this approach enabled us to conduct a comprehensive exploration of the structure-function relationship. The selected apical motifs, namely, _118-_LLF_-120_, _174-_HSGGL_-178_, and _107-_KRTIQKK_-113_, were chosen based on their distinctive localization within the apical domain, which represents the most divergent structural elements among the neuraminyllactose-binding hemagglutinin protein family. This family of secreted proteins is exclusively found in *Helicobacteraceae* and is believed to facilitate host-pathogen interactions. Evolutionary and structural analysis of the neuraminyllactose-binding hemagglutinin protein family indicates that the apical domain has undergone strong structural and sequence divergences, likely to serve alternative functions or engage with different specific receptors. By introducing charge and hydrophobic reversal mutations, we confirmed the role of the hydrophobic _118-_LLF_-120_ loop motif as a significant contributor to the cell adhesion activity. The mutation led to a substantial 55% reduction in cell adhesion function when tested on gastric epithelial cells. Additionally, we confirmed that the _107-_KRTIQKK_-113_ motif, previously implicated in host-cell interactions, also functions as a binding determinant. This motif comprises positively charged lysine and arginine residues, which likely contribute to critical electrostatic interactions for host-cell recognition. Interestingly, cumulative mutations in both the _118-_LLF_-120_ and _107-_KRTIQKK_-113_ sites aggravated the phenotype with further impaired binding as they constitute a large binding interface on the apical domain of HpaA.

To explore the ability of HpaA to modulate the host cell response, we evaluated the expression of proinflammatory cytokines in macrophages and AGS cells upon interaction with the adhesin. While no significant changes were observed in the cytokines expression in AGS cells, treatment of THP-1-derived macrophages with HpaA resulted in a significant threefold increase TNF-α mRNA expression, suggesting a role for HpaA in promoting proinflammatory responses in macrophages. This finding indicates that HpaA, like other *H. pylori* effectors, can influence host cell responses and cytokine production, which is a common pathogenic trait of *H. pylori* to facilitate colonization of the gastric mucosa by exploiting the pro-inflammatory response (39). The *in vitro* induction of TNF-α by HpaA may represent a novel pathological function of the surface lipoprotein. This is significant considering the well-established link between excessive TNF-α production during chronic *H. pylori* infections and its contribution to gastric tumorigenesis and disruption of the gastric mucosal barrier (48–50). Hence, HpaA may introduce a redundant alternative to the function of the *H. pylori* Tipα virulence factor secreted by the pathogen to ensure TNF-α stimulation through the activation of nuclear factor-κB (NF- κB) (51).

Due to the high conservation of HpaA across various *H. pylori* isolates and its essential role as a surface lipoprotein in bacterial colonization (17, 52), HpaA has emerged as a promising antigen for therapeutic vaccines. The protein has already demonstrated success in rodent vaccine models of *H. pylori* infections using conjugated peptide epitopes or full-length recombinant antigens. Given the relevance of the HpaA structure for vaccine development, we employed this structure to map the most immunoreactive epitopes identified in T-cells from *H. pylori*-infected patients, and to predict putative epitopes as a tool to guide the design of improved immunogens. To achieve this, we employed a structure-based geometric deep learning model (ScanNet) for the prediction of B-cell epitopes to identify novel epitopes for vaccine development (42, 53). B-cell epitopes play a critical role in mounting rapid and robust antibody responses against pathogens, resulting in immediate protective immunity against the specific antigen, and contributing to the establishment of long-term humoral memory responses (54). We identified two potential B-cell epitopes located onto the protruding loops of the apical domain, which were tested in our structure activity study. Notably, the HpaA_124–139_ predicted epitope overlaps with the functionally important _118-_LLF_-120_ binding motif, which is critical to promote efficient HpaA’s cell adhesion activity. Given the essential role of HpaA in establishing successful colonization in animal models (17, 55), directing the immune response toward the HpaA_124–139_ epitope represents a strategic approach to develop alternative vaccine formulations that would be capable of disrupting an essential *H. pylori* function.

In summary, this study provides valuable insights into the structure, function, and immunogenicity of HpaA. The crystal structure uncovers a conserved structural fold with divergent structural elements in the apical domain that are crucial for HpaA’s adhesion function. Moreover, our study demonstrates the ability of HpaA to induce TNF-α expression in macrophages, highlighting a novel role as an immunoregulatory effector promoting the pro-inflammatory response. Overall, these findings contribute to a better understanding of the role of HpaA in *H. pylori* pathogenesis and provide a foundation for the design of structure-based HpaA derivatives to improve its vaccine efficacy.

## MATERIALS AND METHODS

### Molecular cloning and site-directed mutagenesis

The numbering of residues initiates with the first mature amino acid (Cys-1) remaining after signal peptide cleavage. The region coding the mature HpaA_26–233_ sequence from *H. pylori* 26695 strain was PCR-amplified from genomic DNA and cloned by restriction-free cloning method into a modified pNIC vector encoding a carboxy-terminal hexahistidine purification tag. The resulting recombinant plasmid was transformed into SIG10 competent *Escherichia coli* and cultures in selective LB-agar medium containing 50 µg/mL ampicillin. Resulting positive colonies were grown in selective LB, and the recombinant plasmid was purified prior to sequencing. Plasmids encoding mutant HpaA constructs were produced by site-directed mutation cloning method and subsequently validated by Sanger sequencing. Oligonucleotide sequences for HpaA cloning and site-directed mutagenesis are listed in Table S1.

### Protein expression and purification

For large-scale expression, 50 mL of an overnight culture was used to inoculate 2L of LB media supplemented with 50 µg/mL ampicillin. Expression of the recombinant proteins was induced by the addition of a final concentration of 0.5 mM isopropyl-1-thio-β-d-galactopyranoside when the cell density reached an OD_600_ between 0.6 and 0.8 a.u. After overnight incubation at 20°C, cells were harvested by centrifugation and resuspended in cold purification buffer (20 mM HEPES pH 8.0, 200 mM NaCl) supplemented with 300 µg/mL lysozyme, 4 µM DNAse I, 4 µM MgCl_2_, and 500 µM phenylmethylsulfonyl fluoride prior to sonication cell lysis. Bacterial debris were removed by centrifugation at 15,000 RPM and flow-filtration (0.8 µm pore size). The clarified supernatant was ultimately loaded onto 5 mL Ni-NTA resin and eluted using an imidazole step gradient. The eluted his-tagged proteins were concentrated to 10 mg/mL using a 10 kDa cutoff amicon ultra centrifugal filter and loaded onto a superdex75 size-exclusion column. HpaA_26–233_ ran as a single elution peak in 20 mM HEPES pH 8.0, 200 mM NaCl, which was used as the mobile phase buffer. Purity was verified using SDS-PAGE and recombinant HpaA proteins were concentrated to 20 mg/mL and kept at 4°C for short term use, or alternatively frozen at −20°C for long-term storage.

### Fluorescent protein labeling

All constructions of HpaA_26–233_, Tipα, and BSA were labeled with fivefold molar excess of N-hydroxysuccinimide-rhodamine (Thermofisher) for 2 h at 4°C. The reaction was stopped by adding 100 mM of glycine and unbounded rhodamine was removed by gel filtration chromatography with a superdex 75 10/300 column (Cytivia). Protein concentration and degree of labeling were calculated according to the manufacturer’s instructions.

### Multiangle light scattering

The molecular size and homogeneity of purified HpaA_26–233_ were assessed at 25°C with a Waters BioSample Acquity UPLC separation module equipped with a Wyatts MicroDAWN (MALS) detection system as well as PDA and Optilab UT-rEX detectors to monitor static light scattering, UV, and refractive index, respectively. The samples were loaded with an auto-sampler into the chromatography system and injected onto an Acquity UPLC Protein BEH SEC 200Å column (1.7 µm, 4.6 mm × 150 mm) at a flow rate of 0.4 mL/min. The column was previously equilibrated in the phosphate buffer (137 mM NaCl, 2.7 mM KCl, 8 mM Na_2_HPO_4_, and 2 mM KH_2_H2PO_4_ at pH7.5) used as mobile phase. Molar weight determination was performed with the Astra software (Wyatt Technology) using Herceptin as a calibration standard.

### Differential scanning fluorimetry

Thermal stability of all constructs was measured with 0.1 µg/µL of protein sample in 20 mM HEPES pH 8.0, 200 mM NaCl buffer. The melting temperature was determined on a StepOnePlus Real-Time PCR System by monitoring the fluorescence emission of SYPRO orange dyes reporting the thermal denaturation of protein samples in a 96-well plate as the temperature increases from 25°C to 99°C with increments of 0.3°C per second.

### Crystallization and data collection

The purified HpaA_26–233_ protein was screened with an automated Gryphon-LCP robot (Art Robbins Instruments) against the MCSG, PACT, and JSCG + crystallization suites. Initial HpaA crystals were observed in 0.1 M sodium acetate trihydrate pH 4.5 and 25% PEG 3350 (wt/vol) then optimized with a 1:1 ratio sitting drop at 20°C in a precipitant solution composed of 0.1 M sodium acetate pH 4.5, 23% PEG 3350 (wt/vol), and 6% glycerol (wt/vol) yielding crystals in space group P2_1_. Crystals were cryo-protected in mother liquor supplemented with 30% glycerol (wt/vol) and flash-frozen in liquid nitrogen. Data were gathered on frozen crystals on beamline 08ID-1 at the Canadian Light Source (CLS). Diffraction data sets were collected at a wavelength of 0.95375 Å using 0.1° oscillation recording 3,600 images, which were processed with X-ray diffraction studies (XDS) to 2.9 Å resolution. Data collection and refinement statistics are summarized in Table 1. A partial structural model (∼60% of the protein sequence) was obtained by molecular replacement based on a truncated Alphafold2 model using PHASER (56), yielding an initial *R*_work_/*R*_free_ 0.43/0.44. A poly-alanine model was manually built, and the final model was generated following several rounds of model building and refinement using Coot (57) and Phenix (56) and yielded a final *R*_work_ of 24% and *R*_free_ of 30% with four HpaA molecules in the asymmetric unit. A Ramachandran plot calculated 3.6% of residues in the allowed region and 0.3% as outliers. Coordinates and structure factors have been deposited in the Protein Data Bank under the accession code 8T8D.

### Bioinformatical analysis

The sequences of HpaA homologs were obtained through a PFAM analysis (58) utilizing HpaA (Uniprot accession number P55969) as the protein query sequence, leading to the identification of 2002 proteins within the PFAM entry PF05211. Subsequently, these protein sequences underwent cluster analysis with CLANS (34) using a multiple sequence alignment as input. Structural homologs were identified through the PDBeFold server (33), using PDB entry 8T8D as the query structure. Manual inspection using PyMOL superposition was then conducted to validate the structural homologs. B-cell epitopes were predicted using the ScanNet server (42), employing PDB entry 8T8D as the query structure to pinpoint favorable protein-antibody interaction sites using the antibody prediction mode.

### Cell culture

THP-1 and AGS cells were initially obtained from the American Type Culture Collection (ATCC). AGS cells (a human gastric adenocarcinoma cell line) were maintained in F-12K medium supplemented with 10% fetal bovine serum, 50 µg/mL streptomycin/penicillin at 37°C in a 5% CO_2_ incubator. Macrophages were derived from THP-1 monocyte cells cultured in RPMI 1640 medium supplemented with 10% fetal bovine serum, 1 mM pyruvate, 1× non-essential amino acids, 50 µg/mL streptomycin/penicillin and maintained at 37°C in a 5% CO_2_ incubator. The THP-1 cells were differentiated into macrophages by stimulation with 1 µM phorbol 12-myristate 13-acetate (PMA) for 48 h followed by 24 h incubation in supplemented RPMI 1640 medium.

### Cell adhesion assays

350,000 AGS cells in 200 µL of F12K were incubated with different concentrations of rhodamine-labeled protein (0.5–20 µg/mL) during 0–120 min at 37°C. After three washes with PBS, cells were resuspended in cold PBS and analyzed by flow cytometry (BD LSRFortessa Cell Analyzer). Rhodamine-labeled BSA was used as negative control. Binding of rhodamine-labeled protein to AGS cells was measured as mean fluorescence intensity. Interactions between rhodamine-HpaA (4 µg/mL) and rhodamine-Tipα (20 µg/mL) with AGS cells were also analyzed in the presence of 800 µg/mL unlabeled N-Acetylneuraminic acid for competition assays (Neu5Ac, Sigma Aldrich).

### Cytokine expression

350,000 AGS cells or THP-1-derived macrophages were seeded into 12-well plates 24 h before experimentation. Cells were exposed to 50 µg/mL HpaA, 100 µg/mL Tipα (positive control), 10 µg/mL lipopolysaccharide (LPS) endotoxin (positive control), or an equal volume of buffer effluent from the protein purification column (negative control) added to the culture media for a period of 4 h at 37°C. Endotoxin testing revealed similar trace amount of endotoxins between the negative control and the HpaA wells (3 and 5 ng/mL, respectively). After three washes with PBS, the total RNA fractions were extracted from all samples according to the manufacturer’s instructions (Life Technologies). Two micrograms of RNA, determined by optical density reading at 260 nm, were used in the reverse transcriptase reaction using a LunaScript RT SuperMix Kit (New England BioLabs) according to the manufacturer’s instructions. The integrity of the extracted RNA and cDNA obtained after quantitative RT-PCR was verified by electrophoresis on 1% agarose gel for quality control. Oligonucleotides for the specific detection of human Interleukin-8, Tumor Necrosis Factor-α, Interferon-γ, and glyceraldehyde-3-phosphate dehydrogenase (GAPDH) were purchased from Sigma-Aldrich. PCR amplifications were performed in a 96-well plate using 20 µL reaction volumes containing 2.5 µL of cDNA, 10 µL of Luna Universal qPCR Master Mix (New England BioLabs), and 0.25 µM of each primer. Each PCR amplification was performed in technical triplicate for two biological duplicates in a StepOne Real-Time PCR System (Applied Biosystems), using the following conditions: 95°C for 60 s (initial denaturation), 95°C for 15 s (denaturation), and 60°C for 30 s (annealing/extension) over 45 amplification cycles. The cycle thresholds (*Ct*) were determined for each experiment and then standardized against the *Ct* of the internal GADPH control (endogenous housekeeping gene). The 2^−ΔΔ*C*^t method (59) was used to quantify the expression of IL-8, TNF-α, and INF-γ in each sample compared to their expression in the reference experiment (cells treated with buffer). Experimental results are expressed as an *n*-fold change relative to the reference. All assays were performed with biological duplicates. Oligonucleotide sequences for quantitative RT-PCR are listed in Table S1.

## Data Availability

The structural factors and coordinates of HpaA_26–233_ have been deposited in the Protein Data Bank under the accession code 8T8D.

## SUPPLEMENTAL MATERIAL

Supporting information mbio.02952-23-s0001.pdf Figures S1 to S5 and Table S1.

ASM does not own the copyrights to Supplemental Material that may be linked to, or accessed through, an article. The authors have granted ASM a non-exclusive, world-wide license to publish the Supplemental Material files. Please contact the corresponding author directly for reuse.
