# Supplementary material for: Unraveling the crystal structure of the HpaA adhesin: insights into cell adhesion function and epitope localization of a Helicobacter pylori vaccine candidate
Source: mBio. 2024 Feb 20;15(3):e02952-23. doi: 10.1128/mbio.02952-23 (PMC10936181; doi:10.1128/mbio.02952-23)
Supplement: Supporting information — Figures S1 to S5 and Table S1. [file mbio.02952-23-s0001.pdf]

Supporting information for

Unraveling the Crystal Structure of the HpaA Adhesin: Insights into Cell Adhesion Function and Epitope Localization of a Helicobacter pylori Vaccine Candidate

To whom correspondence should be addressed
E-mail: charles.calmettes@inrs.ca

This PDF file includes:

S1 Fig. Asymmetric unit organisation of the HpaA crystal
S2 Fig. HpaA does not bind to Annexin A2 and glycosylated mucin
S3 Fig. Cell host interaction, focus on the $_{120}$-KRTIQKK-$_{126}$ motif
S4 Fig. Sequence conservation and structural comparison with AlphaFold
S5 Fig. HpaA binds to macrophage cells in vitro
S1 Table. Oligonucleotides for cloning and quantitative RT-PCR

Figure S1

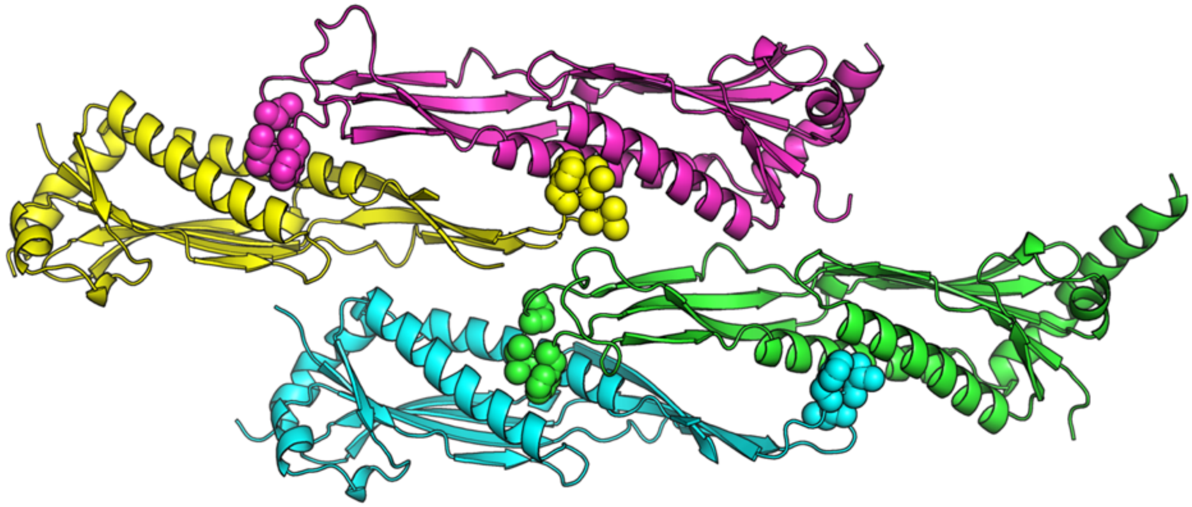

S1 Fig. Asymmetric unit organisation of the HpaA crystal. Illustration of the asymmetric unit from the $HpaA_{26-233}$ crystal depicted in a cartoon representation. The structures are color-coded to highlight individual HpaA molecules, with the side chains of the $_{118}$-LLF-$_{120}$ motif (loop 5) visualized in sphere representation.

Figure S2

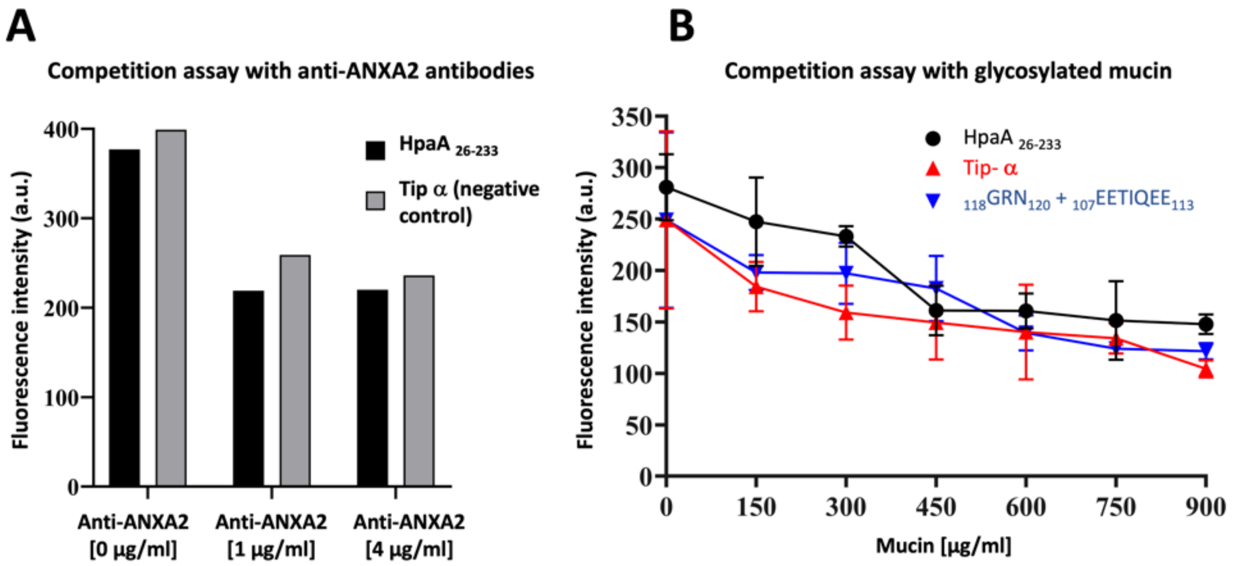

S2 Fig. HpaA does not bind to Annexin A2 and glycosylated mucin. (A) Anti-annexin A2 (anti-ANXA2) competition assay. The histogram describes the binding association of RhB-labeled HpaA and Tipα (negative control) proteins as monitored by flow cytometry in presence and absence of anti-ANXA2 competitors. AGS cells were pre-incubated with anti-ANXA2 antibodies for 30 minutes prior addition of the HpaA/Tipα analytes for an additional 30 minutes at 4°C. Quantification of the cell fluorescence was performed in a flow cytometer. Anti-ANXA2 antibodies do not impede adhesion of HpaA to AGS cells. No significant difference ($p \geq 0.05$; using a two-way ANOVA test followed by Sidak correction) was observed in presence or absence of annexinA2 antibodies in one experiment. **(B)** Competition assay with glycosylated mucin. The graph depicts the binding of fluorescently labelled HpaA wild type and $_{118}$GRN$_{120}$ /$_{107}$EETIQEE$_{113}$ mutant, and Tipα (negative control) proteins to AGS cells, as monitored by flow cytometry with increasing concentration of mucin using biological triplicates. Compared to the Tipα negative control that binds to AGS cells in a nucleolin-dependent manner, glycosylated mucin does not compete for interactions with HpaA wilde type or $_{118}$GRN$_{120}$ /$_{107}$EETIQEE$_{113}$ mutant.

Figure S3

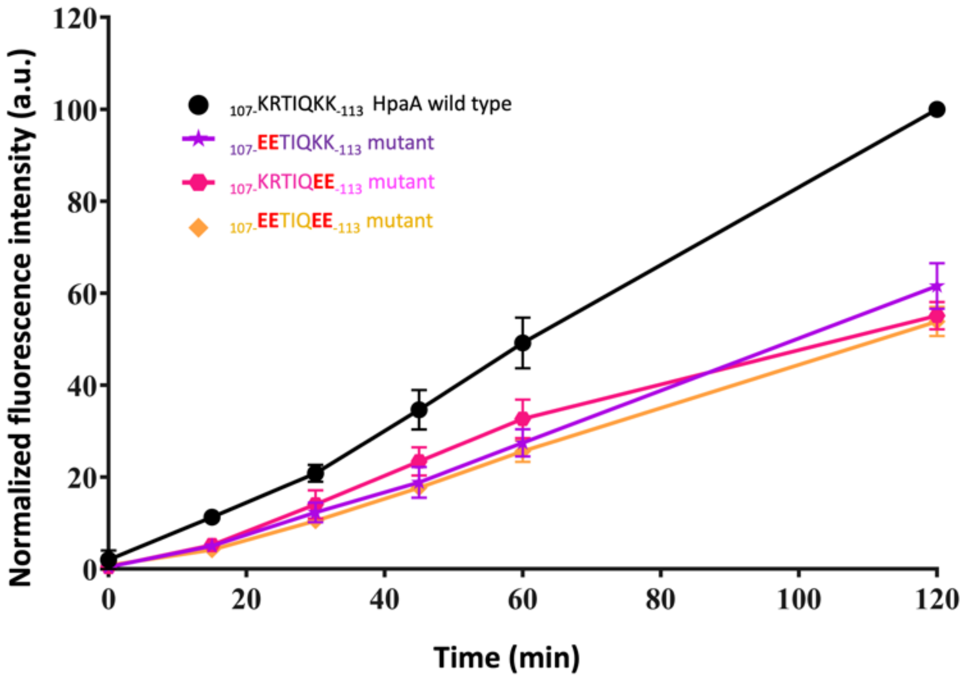

S3 Fig. Cell host interaction, focus on the $_{107}$-KRTIQKK-$_{113}$ motif. The $_{107}$-KRTIQKK-$_{113}$ motif was mutated in EETIQEE. The supplementary figure illustrates the effect of each EETIKK and KRTIQEE mutated sites within the motif according to the cell adhesion function. These data complement the results presented in figure 4C. Both the EETIKK and KRTIQEE sites are equally sufficient to impede HpaA binding activity. The figure represents biological triplicates.

Figure S4

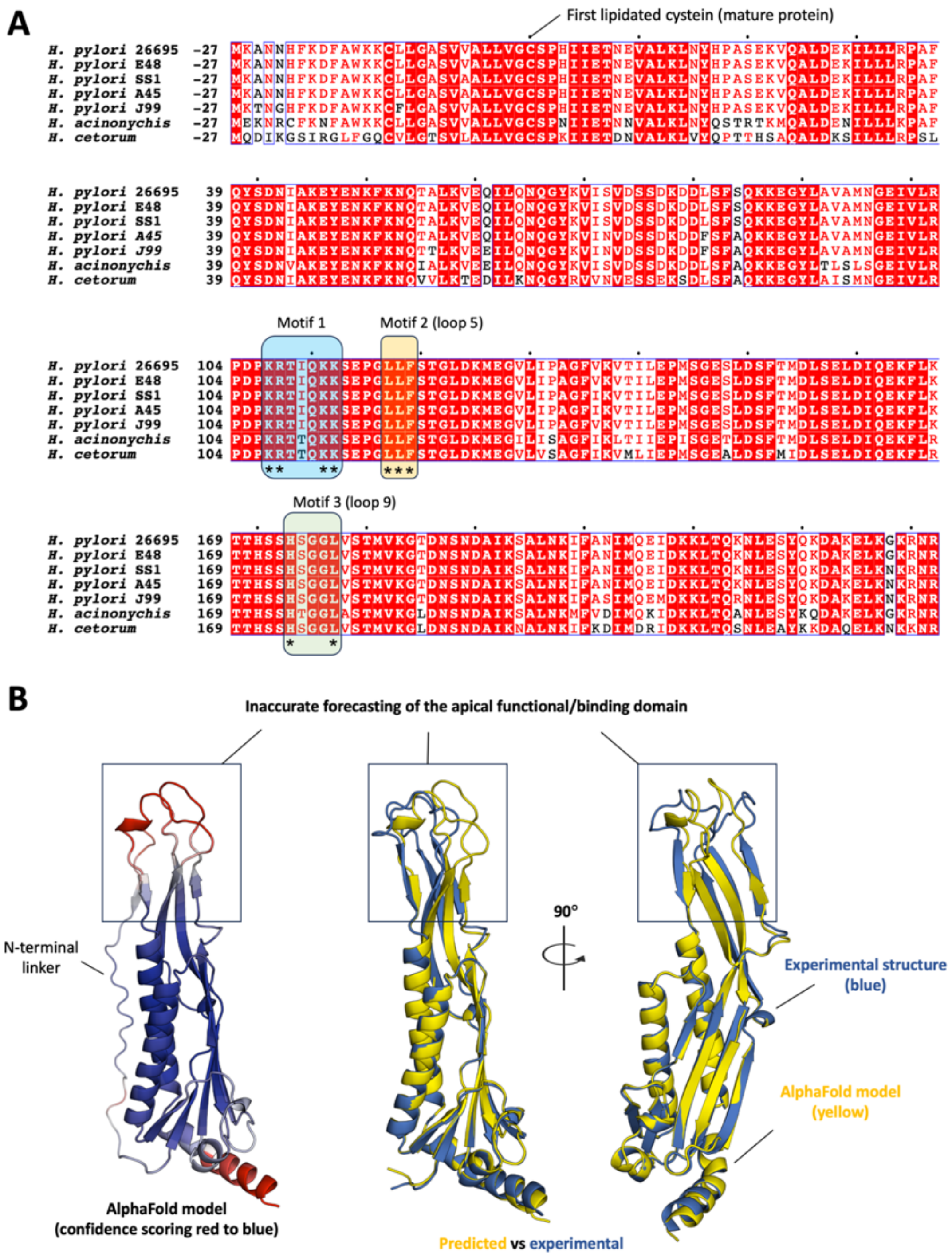

S4 Fig. HpaA sequence conservation among Helicobacteraceae, and structural comparison with the AlphaFold model. (A) Sequence alignment of HpaA across a range of strains, including laboratory *Helicobacter pylori* strains (26695 and J99), clinically isolated strains (E48, SS1, A45), and non-human

colonizers (*H. Acinonychis*, *H. cetorum*), demonstrates robust sequence conservation among species, including the preservation of host-binding motifs. Identical and conserved residues are highlighted in red boxes, and red letters, respectively, while mutation sites are denoted by asterisks. **(B)** Cartoon representation of the HpaA AlphaFold model, color-coded according to confidence scoring (ranging from low confidence in red to high confidence in blue). Comparison with experimental data confirms inaccurate prediction of the apical domain. AlphaFold predicts the flexible amino terminal linker (25 amino acids, absent in the crystallographic structure) to align along the HpaA helice α1, which may represent an alternative conformation that influences the extent to which the globular domain extends away from the membrane, depending on whether it alternates between docked and undocked conformations, as seen in other surface lipoproteins such as TbpB (Calmettes et al. 2011, J. Biol. Chem. doi: 10.1074/jbc.M110.206102). Intrinsically flexible amino-terminal linkers are required for cell surface secretion through the Lol lipoprotein sorting system (El Rayes et al. 2021, Nat. Chem. Biol. doi: 10.1038/s41589-021-00845-z). The AlphaFold model was obtained from the AlphaFold Protein Structure Database entry AF-P55969-F, and displays 100% sequence identity with the experimental HpaA structure solved in this study.

Figure S5

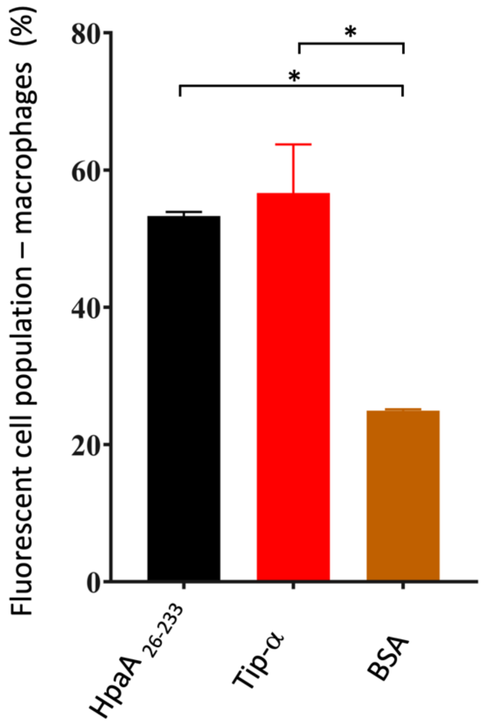

S5 Fig. HpaA binds to macrophage cells in vitro. Histograms depict the binding association of $HpaA_{26\text{-}233}$, Tipα (positive control), and BSA (negative control) rhodamine-coupled proteins with macrophage cells. THP-1 derived macrophages were incubated with 20 µg/ml of $HpaA_{26\text{-}233}$, Tipα, or BSA for 20 minutes, followed by 3 washes prior to detection of cellular fluorescence using flow cytometry. Statistical significance was assessed using biological triplicates (one-way ANOVA test), with asterisks denoting p-values less than 0.001.

Table S1

S1 Table. Oligonucleotides for cloning and quantitative RT-PCR. Oligonucleotides used for HpaA cloning and RT-PCR analyses. Mutated nucleotides are labeled in red.

Oligonucleotides: HpaA cloning and site-directed mutagenesis

$HpaA_{26\text{-}233}$-pNIC-Fwd	gtttaaccttaagaaggagatatactatggcgttagatgaaaagattttgc
$HpaA_{26\text{-}233}$-pNIC-Rev	gatctcagtggtggtggtggtggtgtcggtttcttttgccttttaattc
HpaA-L5mutant-$_{118}$-GRN-$_{120}$-Fwd	cagaaaaaatcagaacccgggggtcgtaactccactggtttggataaaatg
HpaA-L5mutant-$_{118}$-GRN-$_{120}$-Rev	cattttatccaaaccagtggagttacgaccccgggttctgatttttctg
HpaA-L9mutant-H174A/L178R-Fwd	cttaaaaaccacccattcaagcgcgagcgggggcgtgttagcactatggttaagg
HpaA-L9mutant-H174A/L178R-Rev	ccttaaccatagtgctaacacgccccccgctcgcgcttgaatgggtggtttttaag
HpaA -K107E/R107E-Fwd	gaaattgttttacgccccgatcctgaagaaaccatacagaaaaaatcagaac
